# Unveiling the Influence of AI on Advancements in Respiratory Care: Narrative Review

**DOI:** 10.2196/57271

**Published:** 2024-12-20

**Authors:** Mohammed M Alqahtani, Abdullah M M Alanazi, Saleh S Algarni, Hassan Aljohani, Faraj K Alenezi, Tareq F Alotaibi, Mansour Alotaibi, Mobarak K Alqahtani, Mushabbab Alahmari, Khalid S Alwadeai, Saeed M Alghamdi, Mohammed A Almeshari, Turki Faleh Alshammari, Noora Mumenah, Ebtihal Al Harbi, Ziyad F Al Nufaiei, Eyas Alhuthail, Esam Alzahrani, Husam Alahmadi, Abdulaziz Alarifi, Amal Zaidan, Taha T Ismaeil

**Affiliations:** 1 Department of Respiratory Therapy College of Applied Medical Sciences King Saud bin Abdulaziz University for Health Sciences Riyadh Saudi Arabia; 2 King Abdullah International Medical Research Center Riyadh Saudi Arabia; 3 Department of Respiratory Services King Abdulaziz Medical City Ministry of National Guard Health Affairs Riyadh Saudi Arabia; 4 Anesthesia Technology Department College of Applied Medical Sciences King Saud Bin Abdul-Aziz University for Health Sciences Riyadh Saudi Arabia; 5 Department of Physical Therapy Northern Border University Arar Saudi Arabia; 6 Department of Respiratory Therapy College of Applied Medical Sciences University of Bisha Bisha Saudi Arabia; 7 Health and Humanities Research Center University of Bisha Bisha Saudi Arabia; 8 Department of Rehabilitation Science College of Applied Medical Sciences King Saud University Riyadh Saudi Arabia; 9 Clinical Technology Department, Respiratory Care Program Faculty of Applied Medical Sciences Umm Al-Qura University Mekkah Saudi Arabia; 10 Academic and Training Affairs Riyadh Second Health Cluster Riyadh Saudi Arabia; 11 Department of Respiratory Therapy College of Applied Medical Sciences King Saud bin Abdulaziz University for Health Sciences Jeddah Saudi Arabia; 12 King Abdullah International Medical Research Center Jeddah Saudi Arabia; 13 Basic Sciences Department College of Sciences and Health Professions King Saud bin Abdulaziz University for Health Sciences Riyadh Saudi Arabia; 14 Department of Computer Engineering Al-Baha University Alaqiq Saudi Arabia; 15 Department of Respiratory Therapy Faculty of Medical Rehabilitation Sciences King Abdulaziz University Jeddah Saudi Arabia; 16 Department of Public Health College of Public Health and Health Informatics King Saud bin Abdulaziz University for Health Sciences Riyadh Saudi Arabia

**Keywords:** artificial intelligence, AI, respiratory care, machine learning, digital health, narrative review

## Abstract

**Background:**

Artificial intelligence is experiencing rapid growth, with continual innovation and advancements in the health care field.

**Objective:**

This study aims to evaluate the application of artificial intelligence technologies across various domains of respiratory care.

**Methods:**

We conducted a narrative review to examine the latest advancements in the use of artificial intelligence in the field of respiratory care. The search was independently conducted by respiratory care experts, each focusing on their respective scope of practice and area of interest.

**Results:**

This review illuminates the diverse applications of artificial intelligence, highlighting its use in areas associated with respiratory care. Artificial intelligence is harnessed across various areas in this field, including pulmonary diagnostics, respiratory care research, critical care or mechanical ventilation, pulmonary rehabilitation, telehealth, public health or health promotion, sleep clinics, home care, smoking or vaping behavior, and neonates and pediatrics. With its multifaceted utility, artificial intelligence can enhance the field of respiratory care, potentially leading to superior health outcomes for individuals under this extensive umbrella.

**Conclusions:**

As artificial intelligence advances, elevating academic standards in the respiratory care profession becomes imperative, allowing practitioners to contribute to research and understand artificial intelligence’s impact on respiratory care. The permanent integration of artificial intelligence into respiratory care creates the need for respiratory therapists to positively influence its progression. By participating in artificial intelligence development, respiratory therapists can augment their clinical capabilities, knowledge, and patient outcomes.

## Introduction

### Background

Artificial intelligence is a sophisticated branch of computer science and engineering endowed with the capability to perform intricate data analysis, demonstrating a computational understanding of intelligent behavior [1]. Alan Turing first introduced the concept of simulating intelligent behavior with computers in 1950, including a test to assess whether machines can imitate human intelligence. Later, John McCarthy defined “artificial intelligence” as creating intelligent machines [2]. Artificial intelligence has evolved from simple rules to complex algorithms similar to human intelligence, with various specializations such as machine learning (ML), which uses patterns for decision-making and can be applied dynamically to customize patient care. Moreover, ML has progressed into a more complex form known as deep learning (DL), which uses algorithms to construct artificial neural networks (ANNs) [2]. These ANNs can independently learn and make decisions, emulating human brain functionality. On the other hand, computer vision enables a computer to interpret and comprehend data from images or videos [2]. It is notable that avoidable medical errors are the third leading cause of death in the United States, causing >400,000 deaths annually [3]. Therefore, integrating the use of artificial intelligence into the health care field may have strong positive outcomes [4].

Artificial intelligence pertains to computer systems that have the ability to carry out tasks that are typically exclusive to human intelligence, including visual and speech recognition, decision-making, and language translation [5]. Artificial intelligence systems use techniques such as ML and DL to learn from multiple layers of digital data. ML is a subset of artificial intelligence that involves teaching computer algorithms to acquire knowledge from information and make forecasts or choices without being directly programmed [5]. DL is a subset of ML, which involves training deep neural networks to learn from large datasets that consist of >3 layers and has been particularly successful in tasks such as image and speech recognition [5]. Supervised learning, or supervised ML, involves teaching an algorithm how to map inputs to outputs based on labeled training data, whereas unsupervised learning involves discovering patterns and relationships from unlabeled data [4,6]. Reinforcement learning, where an agent learns by making decisions based on feedback from its environment, has been applied in video game playing or robotics [5,7]. Similar applications can be drawn or applied to the field of medicine [4].

In fact, various artificial intelligence methods, including fuzzy expert systems, Bayesian networks, ANNs, and combined intelligent systems, have been implemented across various medical environments in health care. Specifically, artificial intelligence applications are crucial in health care as they assist clinicians in delivering comprehensive health care services to diverse patient populations with various health conditions [4,8].

In this paper, we conduct a narrative review and provide an in-depth summary of the latest advancements in artificial intelligence as it applies to various scopes of practice within the field of respiratory care. Furthermore, we provide a detailed discussion about the substantial benefits that accompany the implementation and integration of such tools within respiratory care methodologies for clinical purposes. Within this context, we evaluate the progression and application of artificial intelligence, highlighting its relevance across various areas within the field of respiratory care. Experts from various disciplines, based on their areas of expertise and research backgrounds, provided insights and summaries regarding the recent emergence and potential use of artificial intelligence in different aspects of respiratory care: pulmonary diagnostics, respiratory care research, critical care and mechanical ventilation, pulmonary rehabilitation, telehealth, public health and health promotion, sleep clinics, home care, smoking or vaping behavior, and neonates and pediatrics.

### Objectives

The primary aim of this review was to synthesize existing research and expert opinions to assess the current and potential impacts of artificial intelligence technologies on respiratory care practices.

## Methods

This is a narrative review aimed at capturing the broad spectrum of artificial intelligence applications within respiratory care. The focus was on identifying artificial intelligence’s contributions across several key areas (eg, pulmonary diagnostics, respiratory care research, and critical care or mechanical ventilation).

To ensure comprehensive coverage, each domain was independently explored, with the MEDLINE database serving as the primary source of literature; a narrative literature search was performed in MEDLINE and Embase databases via the Ovid portal to select studies that met the inclusion criteria. We included studies that were published in English with full text available, without restrictions on publication date. This review focused on articles discussing the application of artificial intelligence in key respiratory care areas as indicated in their titles or abstracts. Multimedia Appendix 1 provides more information regarding the search terms.

## Results

This review identified key artificial intelligence applications in respiratory care. Each of the following sections highlights artificial intelligence’s role and potential advancements in these aspects of respiratory care.

### Artificial Intelligence in Pulmonary Diagnostics

Advanced technologies such as artificial intelligence, precise ML, and DL have been proposed and harnessed for an array of clinical applications in respiratory care, notably in the realms of pulmonary imaging and pulmonary function tests (PFTs) [9-11]. In addition, certain clinical, ethical, and usability challenges are inherently associated with the implementation of artificial intelligence in the sphere of respiratory care [12].

#### Artificial Intelligence Applications in Chest Imaging

Artificial intelligence applications are frequently used in conjunction with chest x-rays, computed tomography (CT), and magnetic resonance imaging. By leveraging artificial intelligence, we can analyze specific regions in these images, calculate volumes, and extract features with remarkable speed and accuracy. When integrated with ML models, artificial intelligence can assist greatly in quantifying and categorizing image characteristics. For respiratory care practitioners (RCPs), chest x-rays are an essential tool commonly used in critical settings to screen for and interpret pulmonary pathologies. ML models have been used to evaluate chest x-rays, particularly in the diagnosis of COVID-19. For instance, a previous review reported that ML models were used to predict the prognosis of patients with COVID-19 based on chest x-ray analysis [13]. In addition, another ML model demonstrated commendable performance, with a sensitivity of 78% (95% CI 74%-81%) and a specificity of 82% (95% CI 78%-85%) [14]. This highlights the tremendous potential of artificial intelligence in assessing x-rays, enabling clinicians to make evidence-based decisions, and offering a cost-effective solution.

#### Artificial Intelligence Applications in PFTs

PFTs represents one of the crucial responsibilities of RCPs, requiring appropriate training to yield reliable results. Indeed, effective coaching for RCPs is necessary to proficiently administer standard PFTs and derive key indicators, such as the forced vital capacity (FVC), forced expiratory volume in the first second, and forced expiratory volume in the first second/FVC ratio. Moreover, these indexes are evaluated and reported using percentage predicted values, with abnormalities defined by fixed cutoff values. It is important to note that these values have been reported to be influenced by variables such as age and height [15]. Consequently, as PFTs can be reported numerically and reference values are widely available for numerous testing methodologies, ML models could serve as effective tools for evaluating lung function tests. This assertion is supported by a previous multicenter study, which compared a trained ML model with pulmonologists. The ML model was found to outperform pulmonologists in detecting physiological patterns and diagnosing pulmonary diseases [16]. This exemplifies a compelling application of ML to assist clinicians in optimizing diagnostic accuracy through the use of ML-trained data. In addition, ML models have been used to detect poor FVC maneuvers [17]. This could significantly aid practitioners in smaller, rural, or nonspecialist clinics by enabling them to evaluate the maneuver before interpreting the results. Notably, it has been previously highlighted that spirometry maneuvers in general practitioners’ clinics tend to be inaccurate [18].

Despite the availability of robust reference ranges for various ethnic and age groups, spirometry remains underused [19,20]. This deficiency may also be attributed to a lack of trained RCPs to administer this test. By using ML models trained to identify pulmonary pathologies, a cost-effective health care model for pulmonary care can be established, thus aiding in evidence-based clinical decision-making. Indeed, objective physiological pulmonary tests were reported to be underused in advanced health care services [20].

#### Artificial Intelligence Applications in Pulmonary Disease Management

Artificial intelligence and innovative technology offer various ways to enhance personal adherence to therapy, with smart inhalers being a prime example [21,22], and allow for early detection of worsening in chronic pulmonary disease [23,24]. Furthermore, wearable biomedical sensors, for example, can measure physiological parameters and assist in identifying cases of asthma exacerbations and symptoms of worsening chronic obstructive pulmonary disease (COPD) episodes [23,25]. Wearable sensors can also identify environmental parameters, which can be used to create air quality maps, benefiting patients with atopic asthma [26]. Moreover, in the case of COPD, scattered data from PFTs and other medical investigations have enabled the generation of accurate COPD diagnoses and minimized misdiagnoses of asthma as both conditions share symptoms, such as airflow limitations [27,28]. These examples highlight personalized care that focuses on individualized traits to enhance respiratory disease management and alleviate its burden.

In summary, artificial intelligence offers significant potential in pulmonary diagnostics, enhancing accuracy in chest imaging and PFTs. While it improves decision-making and efficiency, challenges such as ethical concerns and the need for trained practitioners remain.

### Artificial Intelligence in Respiratory Care Research

#### Overview

The field of artificial intelligence is rapidly evolving, driven by continuous innovation and research advancements. As a result, the applications of artificial intelligence and ML have expanded to include diverse areas, such as statistical analysis, literature searches, and manuscript preparation for publication [29,30]. In this context, we compiled and analyzed evidence pertaining to artificial intelligence applications in respiratory care research.

#### Applications of Artificial Intelligence in Respiratory Care Research

Artificial intelligence is used to gather, store, access, and analyze data, including large and complex datasets [31,32]. It has proven effective in analyzing data that might otherwise be too time-consuming or require hiring external experts and analysts to evaluate [31]. Furthermore, researchers continue to develop new ML techniques for various applications in scientific research. These techniques help convert raw data into valuable insights, make predictions, categorize information, and enable highly informed decision-making through innovative approaches [33]. Researchers have also adopted novel technologies such as blockchain to support artificial intelligence research, particularly in critical fields such as medicine [34]. Finally, the rapid growth of artificial intelligence and ML in respiratory care research makes it challenging to provide a comprehensive overview of these evolving technologies.

#### Functions of Artificial Intelligence in Respiratory Care Research

##### Analysis

Artificial intelligence enables faster data analysis compared to traditional methods, which typically rely on human-driven sequential procedures for data checking, cleaning, and analysis. Artificial intelligence also enhances the efficiency of filtering and extracting information from datasets [31]. Moreover, artificial intelligence can provide highly accurate results, increasing the reliability of the data and the overall rigor of the research. These technologies are less prone to human errors, resulting in a lower margin of error than human analysis [31].

##### Rapid Advancements

ML software, applications, and techniques gather information and adjust their operations to improve performance on a given task. For example, search engines use user interactions and search histories to deliver results tailored to the user’s specific interests. These ML techniques allow artificial intelligence to continuously improve at an accelerated pace [2].

##### Data Handling

This is a crucial aspect of the research process. Effective data gathering, storage, and accessibility are invaluable tools for researchers. The researchers recognize the importance of cleaning the data before conducting analysis. Artificial intelligence uses various tools and approaches to safeguard collected data from unauthorized access and ensure their long-term storage without accidental tampering, preserving them for future use [31].

#### Role of Artificial Intelligence in Respiratory Care Research

##### Decision-Making

Artificial intelligence is a unique and exciting field within ML that seeks to bridge the gap between humans and machines. This technology focuses on improving how artificial intelligence clearly explains and translates information to users, enabling them to make more informed decisions [35]. Advancements in this technology enable machines to more easily predict the outcomes of actions and decisions for users. Consequently, researchers may use artificial intelligence to assess parameters before making decisions, ultimately enhancing the quality of their research.

##### Imaging

Artificial intelligence has improved the capture and analysis of different types of images [36]. This can further identify pathologies and remarks of various respiratory disease by providing accurate and consistent information. These images can be used for clinical and research purposes.

##### Diagnosis

Researchers have validated the high accuracy of artificial intelligence in diagnosing various respiratory conditions and diseases [37]. These technologies can reduce health care providers’ workloads without compromising the quality of care. Such achievements are acknowledged by the scientific community for enhancing the incorporation of artificial intelligence within the health care sector.

In summary, artificial intelligence represents the next frontier in respiratory care and medical research. While artificial intelligence has already made significant progress, its future promises even greater potential, particularly in areas such as generalization, understanding, data efficiency, transparency, ethical decision-making, emotional intelligence, robustness, and real-time learning. These developments will continue to enhance research and problem-solving across various fields.

### Artificial Intelligence in Critical Care or Mechanical Ventilation

The field of critical care medicine is experiencing significant transformations with the integration of artificial intelligence technologies. Artificial intelligence has proven effective in predicting various clinical outcomes, such as sepsis [38], circulatory failure [39], and mortality rates [40]. Since the 1970s, artificial intelligence and ML have played a pivotal role in mechanical ventilation. An early application, the ventilator manager monitoring system [41], assists clinicians by summarizing the patient’s physiological status, detecting adverse events, suggesting corrective actions, and recommending adjustments to ventilatory therapy based on long-term assessments. It also identifies measurement errors and helps maintain patient-specific goals for ongoing evaluation. Another example, the VQ-ATTENDING system [42], evaluates and provides feedback on ventilator settings to ensure their appropriateness. Furthermore, Ganzert et al [43] emphasized that ML and data-mining techniques provide an objective means to analyze the pressure-volume loop obtained through various methods, enhancing clinical decision-making in critical care environments.

Many mechanical ventilation modes do not strictly use artificial intelligence or traditional ML. Instead, they are primarily closed-loop ventilation modes, such as automated ventilation, that use advanced feedback control systems. These systems automatically adjust ventilator settings based on the patient’s pulmonary mechanics, using complex mathematical models to optimize respiratory support [44]. A detailed summary of these automated ventilation algorithms is presented in Table 1 [45]. The significant advancements in software and the power of multi-microprocessors have facilitated an increase in the variety and complexity of ventilation modes [44,45]. Currently, there are almost 500 different names for commercial modes derived from 55 distinct ventilators. However, a taxonomy classification reveals that only 74 of these modes are truly unique [46]. Recent data [44] demonstrate that, in patients with moderate to severe acute respiratory distress, the closed-loop ventilation mode, specifically INTELLiVENT–adaptive support ventilation (ASV), resulted in lower transpulmonary driving pressures, inspiratory pressures, and respiratory rates while increasing tidal volume compared to conventional ventilation. INTELLiVENT-ASV automatically sets and adjusts tidal volume, respiratory rate, positive end-expiratory pressure, and the fraction of inspired oxygen to minimize the work and force of breathing. This mode operates based on measured physiological parameters such as end-tidal carbon dioxide and oxygenation saturation, as well as user-defined maximum limits (eg, maximum pressure limit) [45]. Another randomized controlled trial by De Bie et al [47] indicated that, in patients undergoing cardiac surgery, INTELLiVENT-ASV led to 30% more time spent using optimal ventilation settings, 2.5% less exposure time to injurious ventilation, significantly reduced risk of severe hypoxemia (risk ratio=0.26, 95% CI 0.22-0.31), and faster assumption of spontaneous breathing (hazard ratio=1.38, 95% CI 1.05-1.83) compared to conventional ventilation.

Artificial intelligence has the potential to facilitate individualized mechanical ventilation by using comprehensive patient and population data alongside continuous monitoring. A recent systematic review exploring artificial intelligence applications in mechanical ventilation identified its most prevalent uses: predicting weaning success, initiating mechanical ventilation, detecting ventilation complications, and recognizing patient-ventilator asynchrony. However, most of the studies in this review exhibited significant bias, and as they were retrospective single-center studies, they suffered from limited external validity [48]. Moreover, extensive research has used computational modeling to provide a mechanistic understanding of respiratory pathophysiology [49,50] and optimize ventilator settings [51-53]. These studies underscore the depth and range of analytical strategies that can enhance the efficacy and personalization of mechanical ventilation.

The Better Care system is not a ventilation mode but a closed-loop monitoring system that uses an algorithm to continuously assess a patient’s respiratory effort. It detects and quantifies ineffective efforts, providing real-time feedback to clinicians. The system’s effectiveness has been validated for accuracy against experienced clinicians, showing high sensitivity and specificity in detecting ineffective respiratory efforts [54]. The implications of these capabilities are significant for patient outcomes. For instance, a study highlighted that desynchronization during mechanical ventilation is common and is associated with increased mortality [55]. In addition, an innovative detection tool within the system has been developed to automatically identify and quantify reverse triggering events, a prevalent issue. This algorithm demonstrated high sensitivity and specificity in comparison to manual detection by 3 experienced clinicians [56].

Despite the potential benefits of artificial intelligence, several limitations warrant careful consideration. One major limitation is the need for large volumes of high-quality data to effectively train artificial intelligence algorithms. In respiratory care, data collection is often time-consuming, and variability in data collection and recording practices can pose significant challenges. Another critical issue is the potential for bias within artificial intelligence algorithms, which can lead to inaccurate predictions or recommendations, as highlighted by Obermeyer et al [57]. It is essential to ensure that the deployment of artificial intelligence technologies adheres to ethical standards and responsible practices to mitigate potential harms or negative consequences. This includes promoting transparency and accountability in artificial intelligence development and use, addressing concerns such as bias and discrimination, protecting individual privacy and security, and ensuring that artificial intelligence technologies contribute positively to humanity [57,58]. Furthermore, it is crucial that respiratory therapists (RTs) are equipped with the necessary skills, training, and knowledge to effectively interact with and interpret the outputs of artificial intelligence algorithms. This training will enable them to integrate artificial intelligence insights into clinical decision-making processes effectively. In brief, the potential of artificial intelligence in mechanical ventilation relies on overcoming challenges such as data quality and algorithmic bias and on ensuring ethical use.

**Table 1:** A summary of some of the currently available algorithms for intensive care unit mechanical ventilators [45].

Algorithm	Description	Benefits	Limitations	Year introduced
PAV+^a^ by Medtronic	Delivers inspiratory assistance proportional to patient effort.	Improves patient-ventilator synchrony, may reduce sedation requirements, and better matches patients’ respiratory drive.	Requires additional monitoring. May not be suitable for patients with unstable respiratory drive, dynamic hyperinflation, or air leak.	1992
ASV^b^ by Hamilton	Automatically regulates tidal volume and respiratory rate based on the set minute ventilation and lung mechanics.	Simplifies ventilator management, improves patient-ventilator synchrony, and reduces time on a ventilator.	May not be suitable for patients with severe lung injury or increased airway resistance.	1998
NAVA^c^ by Maquet	Delivers assistance proportional to patients’ diaphragmatic electrical activity.	Improves patient-ventilator synchrony, may reduce sedation requirements, and allows for more precise and personalized ventilation.	Requires specialized equipment and training. May not be suitable for patients with certain neurological or neuromuscular conditions.	2002
VTV^d^, available from many manufactures	Targets a specific tidal volume via titrating the inspiratory pressure.	Prevents lung injury and simplifies ventilator management.	May not be suitable for patients with unstable lung mechanics, leaks, or varying respiratory demand.	—^e^
Auto-release feature in APRV^f^ by Dräger	The duration of the lower pressure is automatically adjusted to terminate expiration at a certain percentage of peak expiratory flow set by the user.	Creates appropriate intrinsic PEEP^g^ to prevent lung derecruitment, leading to improved oxygenation and reduced VQ mismatch.	May require frequent adjustments and monitoring. More studies are needed to determine its long-term efficacy and safety.	—
Auto-Trak by Philips	NIV^h^ advanced algorithm to optimize patient-ventilator synchrony	Accurate detection of inspiratory efforts while a leak is present, dynamic adaptation to changing patterns, and adjustment of pressure support effectively	Effectiveness varies with patients. May require fine-tuning. Requires skilled health care professional supervision. Influenced by sensor integrity. Not for invasive ventilation.	—
INTELLiVENT-ASV by Hamilton	Automatically adjusts V_T_^i^, RR^j^, PEEP, and FiO_2_^k^ aiming to lower the work and force of breathing.	Personalized and protective ventilation, reduced clinician workload, and a faster weaning process	Complexity, limited data inputs, restricted human judgment, and limited validation in certain populations	2011

^a^PAV+: proportional assist ventilation.

^b^ASV: adaptive support ventilation.

^c^NAVA: neurally adjusted ventilatory assist.

^d^VTV: volume-targeted ventilation.

^e^Not reported.

^f^APRV: airway pressure release ventilation.

^g^PEEP: positive end-expiratory pressure.

^h^NIV: non-invasive ventilation.

^i^V_T_: tidal volume.

^j^RR: respiratory rate.

^k^FiO_2_: fraction of inspired oxygen.

### Artificial Intelligence in Pulmonary Rehabilitation

The advent of technology has ushered in a new era in which DL and ML are increasingly recognized as vital tools for enhancing the accuracy and quality of health care delivery [59]. These technologies have found multiple applications in the medical field, ranging from speech recognition for detecting changes in breathing patterns to the diagnosis of post–COVID-19 condition, cancer, and COPD [60-63]. Moreover, artificial intelligence has been instrumental in developing tailored exercise programs for sports and weightlifting based on extensive data analysis of tracked vitals, exercise patterns, intensity, and physical limitations [64,65]. Exercise is a critical component of rehabilitation in respiratory health care, as noted by Jones et al [66]. The integration of web-based exercise programs has made rehabilitation more accessible, allowing therapists and patients to engage more efficiently [67]. Pulmonary rehabilitation for patients with muscular impairments and injuries or COPD includes using methods such as neuromuscular stimulation of different muscles for exercises and inspiratory muscle training to improve the physical state [68]. These exercises adhere to established standards that guide clinicians in delivering electrical stimulation as part of conventional care. However, artificial intelligence has the potential to transform this domain by offering more precise rehabilitation programs tailored to individual needs after discharge or for those requiring specific respiratory or neuromuscular stimulations. Adjusting pulse amplitude and frequency through artificial intelligence can enable clinicians to prescribe these interventions with reduced effort and ensure uniformity in treatment parameters [69,70].

Nevertheless, artificial intelligence’s role in rehabilitation extends to integrating programs that control neuromuscular stimulation delivery and provide the necessary parameters. This integration could aid in enhancing strength and tolerance through individualized breathing exercises tailored to each patient’s condition. In addition, the care regimen includes both invasive and noninvasive mechanical ventilation to support and manage breathing for patients who are critically ill or chronically dependent, requiring continuous monitoring and adjustments as needed. Deep ML holds significant promise in intensive care units, wards, and outpatient clinics. It can be used to conduct tests (eg, of pulmonary function) to monitor breathing patterns, analyze captured wave data, and recommend or adjust the delivery mode and settings optimally for various situations. This ensures precision and proper interpretation and aims for a more personalized treatment approach, facilitating the rehabilitation process [16,71]. For these reasons, rehabilitation is a crucial phase in any patient’s recovery and management of their condition, significantly influencing their physical, emotional, and social engagement with family, friends, and society [72].

Hence, using artificial intelligence can facilitate access to tailored respiratory or physical exercises devised through the automated analysis of various health markers. These exercises can be personalized using data collected at home via portable devices, smartphones, or wearables, which capture live data and store them in a cloud. This setup allows both practitioners and patients to access the information remotely. Artificial intelligence can also be leveraged to determine the optimal times for medication intake, relaxation, and sleep through continuous monitoring—all crucial for enhancing patient outcomes and improving overall health care delivery. Another promising application of artificial intelligence is the external recording and automated analysis of breathing patterns. This technology can assess breathing variability, adding a vital metric for diagnosing diseases by tracking improvements or deteriorations in respiratory function [73].

In summary, the integration of artificial intelligence into pulmonary rehabilitation represents a significant advancement in health care. Through the use of artificial intelligence for tasks such as neuromuscular stimulation and respiratory function monitoring, this technology has the potential to improve patient outcomes and personalize treatment, setting a new standard for health care and quality of life.

### Artificial Intelligence in Telehealth

**Figure 1:**
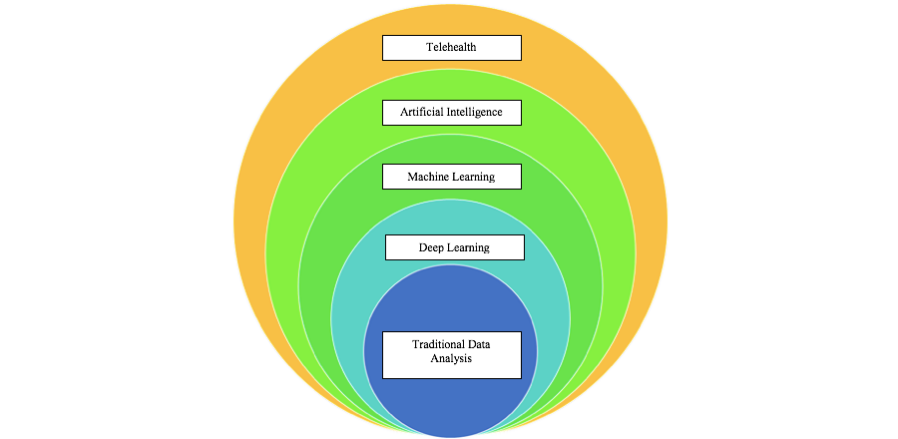
The Telehealth Umbrella Includes Artificial Intelligence, Machine Learning, Deep Learning, and Traditional Data Analysis.

Artificial intelligence represents an approach that falls under the broader category of telehealth, yet it transcends mere use of information and communications technology in supporting health care [74,75]. ML and DL are 2 processes that underpin artificial intelligence, as depicted in Figure 1. Their application in health care is becoming increasingly prominent, enhancing learning, justifying reasoning, and supporting decision-making processes [76,77] Current evidence showcases promising results from the application of artificial intelligence in driving health care innovations [78,79].

Furthermore, a recent systematic review and meta-analysis indicated that this new eHealth approach has gained considerable acceptance among users, proving beneficial in enhancing disease management and quality of life [80,81]. In addition, the ability to detect deterioration and exacerbations in patients with COPD before they occur has been shown to reduce hospital admissions and health care costs [82,83]. In idiopathic pulmonary fibrosis, artificial intelligence has been instrumental in screening radiologic data and simulating accurate diagnoses, thereby saving resources and supporting medical decisions [84,85]. Interpreting results such as cough symptoms through positron emission tomography and CT represents another promising area for artificial intelligence application [86].

In cough analysis, researchers have developed algorithms capable of detecting cough signals, although human input is still essential for refining data and verifying the presence of these events [87,88]. This progress necessitates an enhancement of academic standards for RCPs, enabling them to effectively analyze these signals. Additional applications of artificial intelligence in health care include symptom screening, location detection, infection zone alerts, and the use of robotic surgeons to assist in operations [79,89].

Despite the feasibility and benefits achieved by the application of artificial intelligence in health care, crucial determinants for its operation and sustained use must be identified. Factors such as familiarity with the technology, internet access, data availability, and patient privacy are essential in the ongoing operation and sustainability of artificial intelligence in health care [75,90]. In addition, there is an urgent need to base artificial intelligence applications in health care on a theoretical model that links artificial intelligence content, mechanisms, and outcomes. Addressing current barriers such as lack of knowledge, training, and time requires further research based on real-time clinical data, not just general perceptions about using these telehealth applications [91]. While artificial intelligence may partially or completely transform the health care discipline, standardization and human factors are still necessary for optimal functionality. Artificial intelligence in the clinical setting was initially developed to support health care services and physicians and not intended to replace them. However, as artificial intelligence applications rapidly integrate into health care services, this scenario may change in the coming years. Current efforts should be monitored to minimize errors and establish excellent and sustainable health care services.

### Artificial Intelligence in Public Health and Health Promotion

Due to the explosive growth in health-related data, artificial intelligence, including subfields such as ML, now holds promising potential to enhance public health. Artificial intelligence provides unprecedented insights into social, behavioral, and environmental health determinants. These insights can guide health policy formulation, prioritize focus areas, and foster health improvements across entire populations [92-96]. The substantial advancements in informatics and biotechnology have catalyzed the modernization of various fields, including public health. Precision public health, a contemporary version of public health, uses routinely collected data and artificial intelligence to reinforce existing evidence-based methodologies. The aim is to foster an agile, responsive, and data-driven health care system [97-99].

Artificial intelligence serves as a robust tool for big data analysis in biostatistics and epidemiology. Artificial intelligence algorithms facilitate the detection of intricate patterns in data, which may be challenging for human analysts to discern. These include the identification of disease-specific biomarkers, the prediction of patient outcomes, and the analysis of gene expression data [100,101]. Furthermore, leveraging artificial intelligence in big data analysis not only enhances the explained variance in models but also addresses the shortcomings associated with reliance on a single data source [102].

Moreover, in the realm of health policy and management, artificial intelligence can aid in intricate decision-making and handle complex logistical tasks through real-time information provision. It has been used to tackle health-related challenges, including overcoming the limitations of low- and middle-income countries in achieving the United Nations’ Sustainable Development Goals [103]. Artificial intelligence has also been used in surveillance to visually represent health events geographically and temporally, leveraging public data sources [104], allocation of resources [105], and staffing requirements [106]. In environmental health science, artificial intelligence enhances our understanding of environmental health, analyzes ecological data, and examines the association between environmental factors and health outcomes [107]. In addition, artificial intelligence is extensively used in social and behavioral sciences to deepen our understanding of human behavior through extensive data modeling and investigate correlations among social factors, behavioral factors, and health outcomes. For instance, some studies analyze social media posts to determine the relationship between mental health and social media use, whereas others create models to predict drug use based on a person’s social media activity [108].

Moreover, artificial intelligence can be instrumental in determining the most effective strategies to promote positive health behaviors. This application of artificial intelligence can potentially enable early interventions and lead to improved outcomes in mental health care [109] and chronic disease management and prevention [110]. In addition, artificial intelligence offers innovative ways to facilitate health behavior changes. For instance, it can support smoking cessation programs by using sentiment analysis of X, formerly known as Twitter, data to identify and target individuals most likely to be responsive. This approach leverages artificial intelligence’s capability to analyze vast quantities of data, customizing health interventions to increase their effectiveness [111].

Given the prevalence of respiratory health threats, incorporating artificial intelligence into public health measures for respiratory diseases can lead to more targeted, timely, and population-specific interventions. An example of this is BlueDot, an artificial intelligence–driven algorithm that successfully predicted the early spread of respiratory infections during the initial stages of the COVID-19 outbreak in Wuhan and the Zika virus spread in South Florida. Such applications of artificial intelligence demonstrate significant potential for pre-emptively managing future respiratory health threats [112-114]. Beyond disease prediction, artificial intelligence has numerous other applications in respiratory health. These include developing sophisticated methods for analyzing medical images such as x-rays or CT scans, which can enhance diagnostic accuracy. In addition, artificial intelligence is used to predict disease progression and patient outcomes, providing valuable insights that can guide treatment plans and improve patient care [9,11,84,115,116].

In summary, integrating artificial intelligence into public health and health promotion efforts can significantly enhance respiratory care. By enabling personalized interventions, facilitating early detection of exacerbations, and implementing data-driven strategies, artificial intelligence substantially improves overall respiratory health outcomes from a public health perspective.

### Artificial Intelligence in Sleep Clinics

Artificial intelligence, particularly ML, is increasingly prevalent in sleep medicine. The primary use of artificial intelligence in this field involves classifying sleep stages in polysomnography data capitalizing on ML’s self-learning capabilities [117]. Furthermore, automated polysomnography scoring has been assessed for its potential to augment the efficiency of sleep technologists. By integrating manual reviews with specific computer-derived polysomnography attributes—such as automated sleep spindle detection, sleep depth, and delta duration—interrater reliability has improved. In addition, innovative measures of sleep depth have shown predictive abilities for subsequent arousal [118]. Research on the accuracy of computer-based sleep staging versus human-based scoring has been ongoing since the early 90s. Challenges such as limited sample sizes and the unavailability of sleep-scoring software have historically complicated accurate sleep stage scoring [119]. A study exploring obstacles to artificial intelligence use in sleep medicine identified barriers across technology, data, regulation, human resources, education, and culture, many of which are common to artificial intelligence implementations in various medical diagnostic settings. Thus, addressing these barriers is crucial for enhancing future artificial intelligence applications in the field [120]. ML can significantly enrich polysomnography by unveiling patterns that traditional methods overlook. When combined with clinical and demographic data, it enhances diagnostics and patient care, offering more accurate and comprehensive subtyping of sleep disorders [121]. Although polysomnography is considered the standard screening tool, other questionnaires such as the Berlin Questionnaire, STOP-Bang questionnaire, and Epworth Sleepiness Scale are also widely used. Despite their cost-effectiveness and accessibility, the accuracy of these tools has been questioned [122]. Consequently, recent research has focused on the application of artificial intelligence models for automated scoring and respiratory event detection to enhance the diagnosis of respiratory disorders and more comprehensively capture sleep events [123]. One study assessed the efficacy of generalized regression neural networks in detecting sleep stages, respiratory events, and limb movement in patients with obstructive sleep apnea (OSA), finding that this neural network could predict OSA presence with 98.9% sensitivity and 80% specificity. The aim was to explore whether neural networks could reduce the need for polysomnography in patients without OSA and guide patients with OSA toward treatment rather than diagnostic studies [124]. Another approach used a different neural network with 4 readily available inputs—sex, age, BMI, and snoring status—to demonstrate how an ANN could predict OSA in a clinical setting without the need for oximetry or polysomnography, achieving an accuracy of 86.6% [125]. In addition, a study developed an artificial intelligence–based model using easily accessible questionnaire information to predict sleep apnea, outperforming logistic regression with a sensitivity of 81.8% to 88% and a specificity of 95% to 97% [126].

In summary, artificial intelligence systems could significantly enhance the landscape of sleep diagnostics by addressing traditional limitations, streamlining the diagnostic process, and providing more accurate results, ultimately benefiting patients across various sleep disorders.

### Artificial Intelligence in Home Care

Artificial intelligence has emerged as a crucial technology that enhances the quality of both short- and long-term medical care services, including home care [127,128]. Furthermore, the application of artificial intelligence and ML is anticipated to improve the effectiveness and cost-efficiency of home care as well as enhance patient care overall [129].

An example of artificial intelligence application in home care is the myAirCoach system, which has been effective in managing asthma and improving quality of life among patients with asthma [130]. A study has demonstrated the system’s efficacy in monitoring patients with asthma, leading to reduced severity of asthma exacerbations. However, further research is necessary to fully understand its impact on patient outcomes [130]. The Care Robot represents another artificial intelligence application used to track medication adherence among long-term patients. It alerts caregivers when a patient refuses their medication [131].

The use of artificial intelligence in home care settings is expanding, including the development of an artificial intelligence system designed to assist in assessing and detecting the progression of Parkinson disease (PD) using nocturnal breathing signals during sleep [132]. The application of artificial intelligence in PD evaluation entails multiple benefits. It can reduce the duration and cost of clinical trials; facilitate drug development; and, most importantly, enhance early assessment of PD [132].

Artificial intelligence can be transformative in home care settings, particularly for rehabilitation and palliative care. A study examining home-based rehabilitation using smartwatch and smartphone apps with an ML algorithm for patients requiring physical therapy found that an artificial intelligence–driven home system could enhance outcomes and offer a cost-effective solution for individuals with chronic stroke [133]. In addition, artificial intelligence and ML have been applied for patients with cancer receiving palliative care. Systems designed to assess and monitor medical demands have proven pivotal in managing hospitalization needs. One such system helps palliative care units predict the number of patients requiring hospital admission, thereby improving patient management [134]. A comprehensive system has been developed to assess, monitor, and predict the medical needs and hospitalization requirements of patients with cancer. This artificial intelligence–enhanced system aids palliative care units in managing patient care more effectively by forecasting the need for hospital admissions [134].

In summary, the use of artificial intelligence and ML in home care settings is proving transformative. These technologies not only manage chronic diseases more efficiently but also significantly improve rehabilitation and palliative care processes. Artificial intelligence facilitates a cost-effective approach to enhancing patient outcomes through accurate monitoring and prediction of medical needs. Looking ahead, further advancements in artificial intelligence promise to expand its potential in home health care delivery even more.

### Artificial Intelligence in Smoking or Vaping Behaviors

Both smoking and vaping, the latter involving electronic nicotine delivery systems, represent complex behaviors that pose significant public health threats [135]. Artificial intelligence applications are increasingly being used in the study of smoking and vaping behaviors. These applications use various approaches to predict outcomes related to behavioral factors, treatment efficacy, disease diagnosis, surveillance data, and policy implementation [136-165].

Supervised, unsupervised, and hybrid ML techniques, alongside DL and natural language processing, have demonstrated validity and precision in predicting smoking- or vaping-related outcomes. These advancements could pave the way for personalized care, enhanced cessation services, and more advanced tobacco control implementation and research [136-165].

Consequently, most artificial intelligence applications related to this topic have been deployed to predict smoking or vaping behaviors and their associated factors [136-146,165]. ML and natural language processing are increasingly used to understand smoking or vaping behaviors from various perspectives, including behavioral perception, initiation, continuation, and dependence [137-140,146]. Similarly, various studies have used ML approaches to evaluate mobile apps for smoking or vaping cessation as well as adherence to nicotine replacement therapies [141-143]. In addition, the applicability and accuracy of physiological and wearable sensors in monitoring smoking behavior have been validated using a support vector ML approach [144-146].

It is noteworthy that numerous studies have used smoker data to aid in disease diagnosis and staging predictions. DL and data from chest radiographs and CTs have been effectively used for lung cancer screening. This method has successfully identified high-risk smokers and ascertained their disease stages, predicted prognoses, and estimated mortality rates [147-149]. In addition, gene expression studies involving smokers—which focus on lung cancer and COPD—have been conducted using ML, specifically using the support vector machine method [150,151]. Furthermore, the extreme gradient boosting method has been used to predict noncommunicable diseases induced by smoking through analyzing extensive self-reported datasets [152].

Several artificial intelligence approaches have been used to develop surveillance and registry systems for smokers, as well as to identify public health support and tobacco control threats on social media. Using natural language processing, numerous studies have mined electronic medical and dental records along with medication data to track tobacco use, assess documentation quality, and study longitudinal tobacco use and related health issues. This has significantly contributed to the establishment of tobacco use registries and surveillance systems [153-159]. In addition, through the use of DL and ML techniques, various studies have evaluated public health support for health messaging, assessed public health threats to tobacco control measures, processed images for tobacco point-of-sale advertising, and analyzed smoking locations and environments [111,160-165].

In summary, the application of artificial intelligence in assessing smoking or vaping outcomes has proven both innovative and effective. Given the complexity of these behaviors, sophisticated tools such as artificial intelligence provide a new lens for understanding, preventing, and treating them. The promising potential of artificial intelligence applications in this field warrants further investment, aiming ultimately to reduce the health complications associated with smoking and vaping.

### Artificial Intelligence in Neonates and Pediatrics

The common practice of managing or detecting clinical deterioration in neonatal and pediatric settings primarily depends on the competency of health care providers and the experiences of parents. However, technological advancements in patient assessment and monitoring within these settings present significant opportunities for artificial intelligence application [166].

Pediatric and neonatal diseases often display less heterogeneous patterns and generally have more complete records compared to adult conditions, factors that significantly enhance the potential of artificial intelligence applications in pediatric and neonatal health care [167]. In addition, the practice of admitting neonates from birth enables focused monitoring of physiological parameters and other clinical variables from the outset. Current research on artificial intelligence–based applications for pediatric respiratory conditions primarily focuses on 3 domains: breath sound analysis, chest imaging interpretation, and PFT analysis [168].

Unfortunately, pneumonia remains one of the leading causes of morbidity and mortality in the pediatric population [169]. CAD4Kids is a computer-aided diagnosis system specifically designed to assess childhood pneumonia using chest radiographs as a diagnostic tool [170]. Developed to classify chest x-rays into 3 categories—pneumonia, another infiltrate, or no infiltrate—the artificial intelligence algorithm achieved an impressive area under the curve (AUC) of 0.85, demonstrating a sensitivity of 76% and a specificity of 80% in correctly identifying radiographs with pneumonia [169,170].

In addition, a predictive algorithm using pediatric patients’ temperature, respiratory rate, heart rate, and oxygen saturation has been used to diagnose pneumonia. This algorithm achieved an impressive AUC of 97.8% with a sensitivity of 96.6% and a specificity of 96.4%, showcasing the potential of artificial intelligence in providing accurate pneumonia diagnoses [171]. The simplicity of this algorithm makes it ideally suited for integration into smartphones or tablets, giving health care providers convenient access. This user-friendly approach has the potential to significantly enhance diagnostic capabilities and improve respiratory care for pediatric patients on a broader scale [171].

Croup, a pediatric condition known for its distinctive loud barking cough, has been the focus of a new algorithm developed to identify affected pediatric patients by analyzing the acoustic features of their coughs [172]. This algorithm demonstrated a sensitivity of 92% and specificity of 85%, proving effective in accurately diagnosing croup. In addition, a study focused on asthma control used nocturnal cough frequency as a valid marker, monitoring both audio data and abdominal movements [173]. The findings revealed a significant increase in cough count among children with asthma, underscoring the potential for developing a cough monitoring system. Such a system could assess the level of asthma control and facilitate the implementation of more effective management strategies [173].

In another advancement, a DL model has been trained to predict uncontrolled asthma a week in advance by leveraging a self-monitoring tool and incorporating patient demographics. This innovative approach promises to enhance asthma management by facilitating early intervention and personalized care [174]. The DL model achieved an AUC of 0.75, with a sensitivity of 73.8% and a specificity of 71.4%, effectively predicting uncontrolled asthma a week in advance by using a self-monitoring tool and patient demographics. The potential further development of this model promises to provide early warnings for deteriorating asthma control, enabling timely interventions and leading to improved respiratory health outcomes [174].

In neonatal settings, the abundance of data presents a significant opportunity for ML research [175]. A mature artificial intelligence model uses heart rate characteristic monitoring to enhance outcomes for infants with very low birth weight [176]. The study found that neonates monitored using heart rate characteristics showed improved survival rates and required fewer days on mechanical ventilators. This underscores the potential of artificial intelligence applications to optimize care and improve outcomes for vulnerable neonatal populations [176].

A notable example of ML model development for predicting bronchopulmonary dysplasia (BPD) incidence involves using patient clinical and genetic features, as seen in the BPD risk gene sets [177]. This study suggests that a model combining basic clinical risk factors with genetic data can effectively stratify BPD risk in preterm neonates. The field of predicting BPD has seen rapid growth over the last decade, highlighted by the identification of 26 BPD prediction models in 2012 [178]. However, most of these predictors have shown poor performance and limited promise, underscoring a vital need for further extensive research and clinical validation [178].

While artificial intelligence applications in pediatric and neonatal settings offer significant benefits, they also face various challenges that require careful consideration and resolution [179]. Ensuring the quality of the data used to train these systems is crucial, necessitating rigorous assessments to address issues such as small sample sizes, appropriate handling of missing values, and data heterogeneity [179].

In summary, while artificial intelligence promises significant advancements in pediatric and neonatal care, overcoming obstacles related to data quality and training is crucial for fully realizing its potential.

## Discussion

### Principal Findings

Our search was designed to encompass a broad spectrum of studies, reflecting the diverse applications of artificial intelligence technologies aimed at enhancing respiratory health outcomes and advancing the field of respiratory care. In this domain, artificial intelligence approaches have increasingly been deployed as tools for diagnosis, classification, and prediction of mortality outcomes [10]. Notably, artificial intelligence holds significant potential as a valuable asset in supporting RTs by enhancing care delivery and managing respiratory diseases more effectively. However, the successful integration of these technologies necessitates a robust understanding of digital interventions and artificial intelligence principles. It is equally important to promote collaboration with data specialists and computer scientists to address the challenges associated with implementing artificial intelligence in respiratory care [180]. From our review, it appears that artificial intelligence use and application can eventually elevate the overall quality of care by minimizing errors across the various areas that RTs work in.

The use of artificial intelligence in respiratory care involves the processing of large volumes of personal health information, raising significant data privacy concerns. It is crucial to implement robust data protection measures that comply with regulations such as the General Data Protection Regulation and Health Insurance Portability and Accountability Act (HIPAA). These measures should ensure data anonymization and secure data storage and transmission [181]. Furthermore, informed consent is a cornerstone of ethical medical practice and is particularly pertinent in deploying artificial intelligence technologies. Patients must be adequately informed about how their data will be used, the role of artificial intelligence in their care, and any potential risks associated with artificial intelligence decision-making [182]. This process must be transparent and documented, allowing patients to make well-informed decisions about their care [182]. Furthermore, artificial intelligence systems can inadvertently perpetuate or amplify biases present in their training data, which could lead to unequal treatment outcomes across different demographics. It is imperative to assess and mitigate these biases by ensuring diverse and representative datasets. Moreover, continuous monitoring for biased outcomes and periodic model reassessment should be institutionalized to safeguard against discrimination [183]. Through these measures, we can explore the ethical complexities introduced by artificial intelligence in respiratory care, ensuring that these technologies augment health care delivery without compromising ethical standards or patient trust.

### Implications for Future Work

Our study underscores the importance of integrating training and education for RTs and students on recent and relevant artificial intelligence technologies, ensuring their proficiency in using artificial intelligence tools and analyzing artificial intelligence–generated insights within clinical settings. It also highlights the necessity of encouraging interdisciplinary collaboration among RTs, data scientists, researchers, and artificial intelligence developers to enhance the development and implementation of pertinent artificial intelligence applications and research. These collaborations should aim to produce practical, effective, and user-friendly solutions that benefit patients and improve clinical research outcomes. In addition, it is clear that robust ethical guidelines and data protection measures are essential to ensure privacy and compliance with regulations such as the General Data Protection Regulation and HIPAA, safeguarding patient information. Furthermore, we recommend conducting research and validation studies on artificial intelligence applications in the respiratory care field to ensure that artificial intelligence–driven solutions are evidence based, accurate, reliable, and beneficial across various individuals receiving health care from RTs in different clinical settings.

### Limitations

Our study has some limitations. For instance, the literature review conducted across various areas of respiratory care did not adhere to a standardized review process, such as systematic or scoping review, which may introduce potential biases. These biases could stem from selective inclusion of studies, nonsystematic search strategies, or subjective interpretation of findings. Therefore, it is imperative to adopt a well-standardized review methodology for future studies in each specific area of respiratory care to minimize these limitations and ensure more reliable and comprehensive conclusions.

### Conclusions

Artificial intelligence applications should complement rather than replace the decision-making processes of RTs. By serving as a supportive tool, artificial intelligence enables RTs to enhance patient care, disease management, and respiratory health outcomes. As artificial intelligence continues to permeate medicine, it is imperative for RTs to engage actively in its evolution, ensuring that their involvement is central to artificial intelligence development, which is crucial for improving clinical decision-making. Moreover, as artificial intelligence technology advances, it is essential to elevate academic standards within the respiratory care profession, empowering practitioners to contribute effectively to research and grasp the implications of artificial intelligence on respiratory care. The integration of artificial intelligence into health care is irreversible and creates the need for RTs to positively influence its progression. Active participation in artificial intelligence development will enable RTs to expand their clinical capabilities and improve patient outcomes.

## Appendix

Multimedia Appendix 1 Search terms.

## Abbreviations

ANN: artificial neural network
ASV: adaptive support ventilation
AUC: area under the curve
BPD: bronchopulmonary dysplasia
COPD: chronic obstructive pulmonary disease
CT: computed tomography
DL: deep learning
FVC: forced vital capacity
HIPAA: Health Insurance Portability and Accountability Act
ML: machine learning
OSA: obstructive sleep apnea
PD: Parkinson disease
PFT: pulmonary function test
RCP: respiratory care practitioner
RT: respiratory therapist

## Data Availability

Data sharing is not applicable to this paper as no datasets were generated or analyzed during this study.
