# Supplementary material for: Unveiling the Influence of AI on Advancements in Respiratory Care: Narrative Review
Source: Interact J Med Res. 2024 Dec 20;13:e57271. doi: 10.2196/57271 (PMC11699506; doi:10.2196/57271)
Supplement: Multimedia Appendix 1 [file ijmr_v13i1e57271_app1.docx]

**Search terms:**

The search terms and strategy used were; ('Artificial Intelligence*') AND ('Pulmonary diagnostics.ti,ab.' OR 'Respiratory care research.ti,ab.' OR 'critical care.ti,ab.' OR 'mechanical ventilation.ti.ab' OR 'Telehealth.ti,ab.' OR 'pulmonary rehabilitation.ti,ab.' OR 'Sleep clinics.ti,ab.' OR 'Public Health.ti,ab.' OR 'Health promotion.ti,ab.' OR 'Home Care.ti,ab.' OR 'Neonates.ti,ab.' OR 'pediatrics.ti,ab.' OR 'Smoking.ti,ab.' OR 'Vaping Behavior.ti,ab.' AND 'Respiratory Care.ti,ab.' OR 'Respiratory therapy.ti,ab.')."
